# Prognostic value of brain natriuretic peptide in patients with heart failure and reserved left ventricular systolic function

**DOI:** 10.3892/etm.2014.1635

**Published:** 2014-03-27

**Authors:** HUI GONG, XIN WANG, YI LING, YIJUN SHI, HAIMING SHI

**Affiliations:** 1 Division of Cardiology, Department of Medicine, Huashan Hospital of Fudan University, Shanghai 200040, P.R. China; 2 Division of Cardiology, Department of Medicine, Jinshan Hospital of Fudan University, Shanghai 201508, P.R. China

**Keywords:** brain natriuretic peptide, heart failure, left ventricular systolic function, prognosis

## Abstract

Brain natriuretic peptide (BNP) is used as a prognostic biomarker for patients with heart failure (HF) in clinical practice, however, the correlation between BNP levels and the prognosis of HF in patients with reserved left ventricular systolic function (RLVSF) is not clear. Thus, the aim of the present study was to evaluate the added value of BNP in the prognosis of HF patients with RLVSF. Inpatients with cardiovascular disease (mean age, 65.7 years; male, 790; female, 625) admitted to the Division of Cardiology at Jinshan Hospital of Fudan University (Shanghai, China) between June 2006 and December 2009 underwent follow-up examinations. Plasma BNP levels were analyzed and measurements of the left ventricular ejection fraction (LVEF) were performed by echocardiography. Evaluations of the patients with HF were performed according to the New York Heart Association (NYHA) classification system. The duration of the follow-up period ranged between 21 and 63 months (average duration, 35.8 months) and key events included cardiovascular mortality, readmission due to cardiovascular disease or mortality due to other reasons. Survival times decreased with increasing BNP levels in all the follow-up patients (Spearman’s ρ, −0.1877; P<0.0001). Among the 1,415 patients, 1,312 underwent echocardiographic detection. A total of 395 patients with NYHA classes II–IV and a LVEF ≥45% were selected. The incidence of compound endpoint events was significantly higher in the patients that had BNP levels of >100 pg/ml when compared with the patients that had BNP levels of ≤100 pg/ml (37.07 vs. 23.93%; relative risk, 1.55); consequently the survival times were significantly reduced (P=0.0039). A negative correlation was identified between the BNP levels and the survival times in these patients (Spearman’s ρ, −0.1738; P=0.0005). These results indicated that the levels of BNP may be used to predict the prognosis of patients with cardiovascular disease. The prognoses of patients with higher BNP levels were worse compared with the patients with lower BNP levels. Furthermore, significant correlations were confirmed in the HF patients with RLVSF.

## Introduction

Brain natriuretic peptide (BNP) is a 32-amino acid polypeptide containing a 17-amino acid ring structure common to all natriuretic peptides (1). BNP is stored in human cardiac tissue as BNP-32 with a lesser amount of the precursor preproBNP, and in the circulating plasma as BNP-32 and the N-terminal proBNP (NT-proBNP) (2). BNP is a cardiac neurohormone that is secreted into the plasma from the ventricles in response to ventricular volume expansion and pressure overload. BNP levels are useful for the diagnosis of left ventricular (LV) systolic and diastolic dysfunction and have been shown to correlate with the severity and prognosis (3). BNP provides an easy method for the early detection of heart failure (HF), and for assessing the severity of HF and the effectiveness of treatment (4).

A previous study identified that BNP and NT-proBNP are the prognostic importance in patients with HF and with acute coronary syndromes, and both markers have been shown to be strong predictors of morbidity and mortality (5).

Diastolic wall stress has been shown to exhibit a stronger correlation with the levels of NT-proBNP than that of systolic wall stress (6). The estimation of BNP values may be accepted as a fast and reliable blood test in the diagnosis of asymptomatic diastolic dysfunction in patients with hypertension, diabetes and hypertrophic cardiomyopathy (HCM) (7–9). Measurement of BNP levels, which is simple and noninvasive, can be easily and rapidly conducted in emergency departments to guide therapy, follow the response to therapy and predict the exercise capacity of patients (10). However, the role of BNP as a predictor of morbidity and mortality in patients with diastolic dysfunction is unknown. In 2011, a small sample size study (11) hypothesized that the increase in BNP levels over time directly reflected LV diastolic dysfunction and impairment of exercise tolerance. HF patients with reserved left ventricular systolic function (RLVSF) are considered to be patients with diastolic dysfunction. Therefore, the present study was conducted to evaluate the effect of BNP levels on the survival times of HF patients with RLVSF.

## Patients and methods

### Study subjects and procedures

This was an observational study. Consecutive inpatients with cardiovascular disease, admitted to the Division of Cardiology at Jinshan Hospital of Fudan University (Shanghai, China) between June 2006 and December 2009, underwent follow-up examinations. The Ethics Committee of Jinshan Hospital approved the study protocol and written informed consent was obtained from each patient. The patients were classified according to the initial BNP cutoff point of 100 pg/ml. LV systolic dysfunction was defined by an ejection fraction of <45%, while systolic function was considered to be normal when the left ventricular ejection fraction (LVEF) was ≥45%. Evaluations of the patients with HF were performed using the New York Heart Association (NYHA) classification system. HF was defined as NYHA classes II, III or IV (12). Key events included cardiovascular mortality, readmission due to cardiovascular disease or mortality due to any other reasons.

### Measurement of plasma BNP concentration

Blood samples for the analysis of plasma BNP levels were collected at the time of admission and were obtained from the antecubital vein. BNP levels were analyzed using a Triage^®^ BNP test (Biosite Diagnostics, Inc., San Diego, CA, USA), which is a single-use fluorescence immunoassay device designed to determine the concentration of BNP in EDTA-anticoagulated whole blood or plasma specimens. The specimen was added to the sample port of the test device with a transfer pipette that is designed to deliver the appropriate amount of specimen (250 μl) to the test device. Following the addition of the specimen, the device was inserted into the Triage^®^ MeterPro (Biosite Diagnostics, Inc.). The MeterPro was programmed to automatically perform the BNP analysis following the reaction of the sample with the reagents within the BNP device. The reaction and analysis process occurred over ~15 min. BNP analysis was based on the amount of fluorescence that the MeterPro detected within a measurement zone on the device. A greater amount of fluorescence detected by the MeterPro indicated a higher BNP concentration in the specimen.

### Echocardiography

M-mode and two-dimensional images, as well as spectral and color flow Doppler recordings, were obtained by Vivid 7 ultrasound (GE Healthcare, Andover, MA, USA) with Vivid 7 ultrasound operating at 2.0 to 3.5 MHz. The LVEF was calculated from the four-chamber view images using the formula of Simpson’s rule.

### Statistical analysis

Baseline characteristics of the patients are presented as percentages for dichotomous variables and medians with interquartile ranges for continuous variables such as age. The baseline characteristics were compared between the groups using the Wilcoxon rank-sum test for continuous variables and the χ^2^ test for discrete variables. Survival curves were generated by Kaplan-Meier estimates and differences in the survival rates were compared between groups using the log-rank test. The incidence of endpoint events was compared between the groups by means of relative risk. Spearman’s correlation was used to analyze the correlation between the levels of BNP and the survival times of the patients. BNP levels were evaluated by receiver operating characteristic (ROC) and area under the curve (AUC) analyses for predicting the incidence of clinical compound endpoints. To determine the optimal value of specificity and sensitivity, the closest value to the best specificity and sensitivity point on the ROC curve was identified. P<0.05 was considered to indicate a statistically significant difference.

## Results

### Patient characteristics

The study population consisted of 1,415 patients. The mean age was 65.7 years and almost half the patients were male (790/1,415, 55.8%). The duration of the follow-up period ranged between 21 and 63 months (average duration, 35.8 months). Characteristics of the overall patient population are shown in Table I. Risk factors of the patients included hypertension (953/1415, 67.35%), diabetes (291/1415, 32.33%), dyslipidemia (622/1415, 43.96%), renal dysfunction (115/1415, 8.13%; serum creatinine >84 μmol/l in females; serum creatinine >104 μmol/l in males), myocardial infarction (190/1415, 13.53%) and intervention with medication, including β-blockers, calcium antagonists, diuretics, nitrates, antiplatelet agents, statins, angiotensin converting enzyme inhibitors and angiotensin receptor blockers. The numbers of patients with BNP levels of ≤100 and >100 pg/ml were 900 and 515, respectively. A total of 336 endpoint events occurred, including 143 and 193 in the two BNP groups, respectively. Among the 1,415 patients, 1,312 underwent echocardiographic detection at the same time as admission, including 395 (30.11%) patients with NYHA classes II–IV and a LVEF of ≥45% and 123 (9.38%) patients with systolic dysfunction. The incidence of compound endpoint events was significantly higher in the BNP >100 pg/ml group than in the BNP ≤100 pg/ml group (86/232, 37.07 vs. 39/163, 23.93%; relative risk=1.55) in 395 patients with NYHA classes II–IV and a LVEF of ≥45%.

### Survival curves

**Figure 1:**
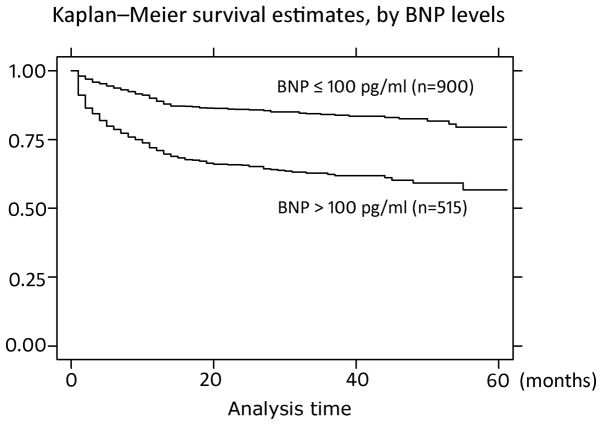
Kaplan-Meier analysis of all the patients with plasma BNP levels higher than 100 pg/ml, and lower than or equal to 100 pg/ml (P<0.0001; χ^2^=94.11). BNP, brain natriuretic peptide.

**Figure 2:**
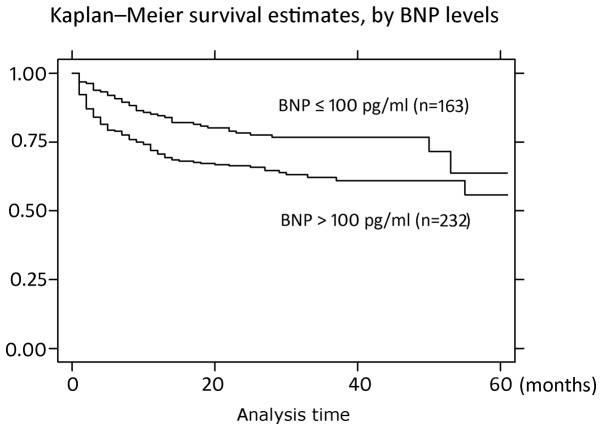
Kaplan-Meier analysis of HF patients with RLVSF with plasma BNP levels higher than 100 pg/ml, and lower than or equal to 100 pg/ml (P=0.0039; χ^2^=8.33). BNP, brain natriuretic peptide; HF, heart failure; RLVSF, reserved left ventricular systolic function.

Kaplan-Meier survival curves of all the patients and specifically the HF patients with RLVSF, according to the BNP levels, are shown in Figs. 1 and 2. The survival curves were constructed in the two groups to predict the survival times of the patients. Survival times were longer in the BNP ≤100 pg/ml group when compared with the BNP >100 pg/ml group and statistically significant differences were observed (P<0.0001, χ^2^=94.11 and P=0.0039, χ^2^=8.33, for all patients and the HF patients with RLVSF, respectively).

### Correlation analysis

**Figure 3:**
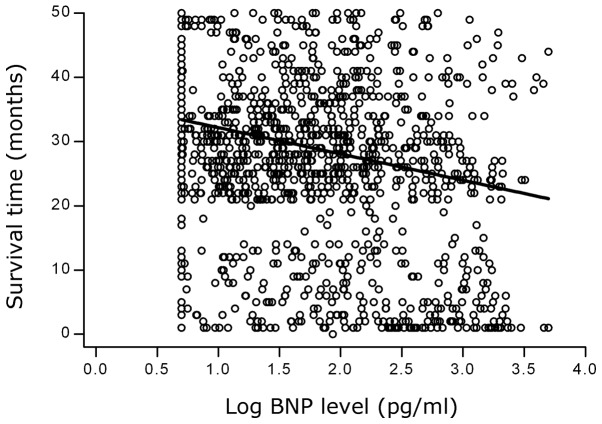
Scatter plot showing the correlation between the logBNP levels and the survival times in all the patients. BNP, brain natriuretic peptide.

**Figure 4:**
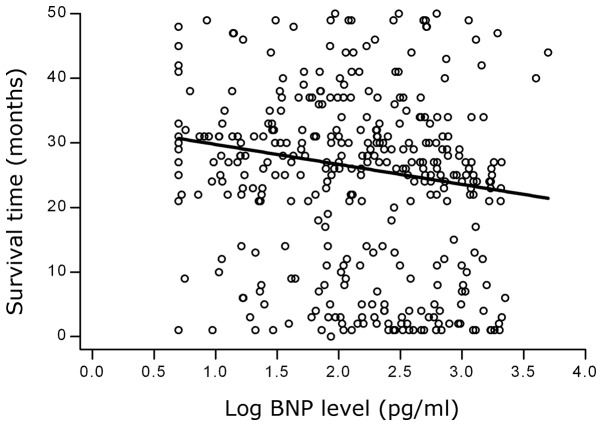
Scatter plot showing the correlation between the logBNP levels and the survival times in the HF patients with RLVSF. BNP, brain natriuretic peptide; HF, heart failure; RLVSF, reserved left ventricular systolic function.

Spearman correlation analysis demonstrated that the survival times decreased as the BNP levels increased (Spearman’s ρ, −0.1877; P=0.0000). A negative correlation between the logBNP levels and the survival times is shown in Fig. 3. A negative correlation was also observed in the 395 HF patients with RLVSF (Spearman’s ρ, −0.1738; P=0.0005). A scatter plot demonstrating the correlation between the logBNP levels and the survival times of the HF patients with RLVSF is shown in Fig. 4.

### BNP levels as a predictor for clinical endpoints

**Figure 5:**
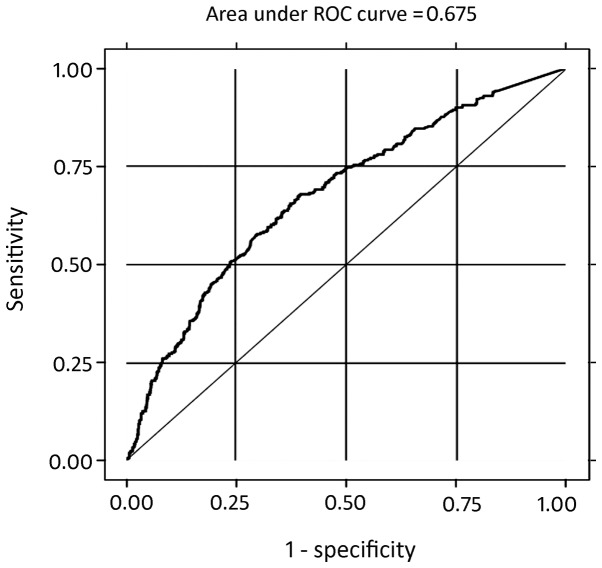
ROC analysis of the logBNP levels for determining the incidence of clinical compound endpoint events in all the patients (AUC, 0.6752; standard error, 0.01698; 95% confidence interval, 0.64198–0.70835). The cut-off value was determined to be 100 pg/ml (specificity, 70.16%; sensitivity, 57.44%). ROC, receiver operating characteristic; BNP, brain natriuretic peptide; AUC, area under curve.

**Figure 6:**
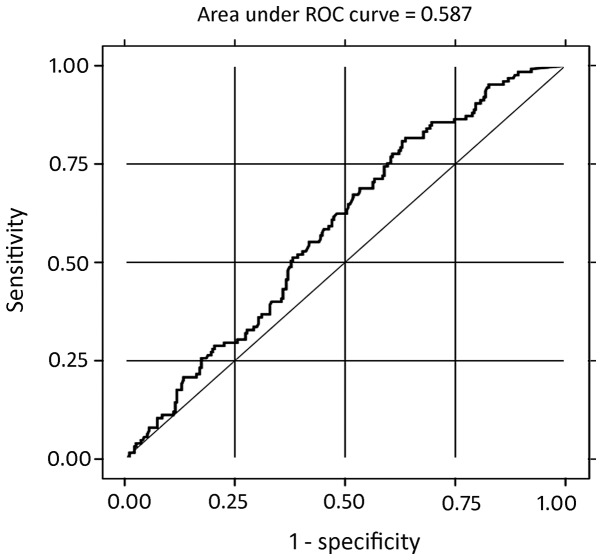
ROC analysis of the logBNP levels for determining the incidence of clinical compound endpoint events in patients with diastolic dysfunction (AUC, 0.5877; standard error, 0.0296; 95% confidence interval, 0.52965–0.64573). The cut-off value was determined to be 100 pg/ml (specificity, 45.93%; sensitivity, 68.8%). ROC, receiver operating characteristic; BNP, brain natriuretic peptide; AUC, area under curve.

The predictive utility of plasma BNP levels in all the patients for determining compound clinical endpoints was calculated with ROC analysis. Plasma BNP levels has diagnostic value in the incidence of clinical compound endpoint events in all the patients and in patients with diastolic dysfunction. The AUROC was 0.6752 with a standard error of 0.01698 (95% confidence interval, 0.64198–0.70835) and the cut-off value for the plasma BNP levels was 100 pg/ml (sensitivity and specificity, 57.44 and 70.16%, respectively; Fig. 5 and Table II). The predictive utility of plasma BNP levels in the HF patients with RLVSF for determining compound clinical endpoints was also calculated with ROC analysis. The AUROC was 0.5877 with a standard error of 0.0296 (95% confidence interval, 0.52965–0.64573) and the cut-off value for the plasma BNP levels was 100 pg/ml (sensitivity and specificity, 68.8 and 45.93%, respectively; Fig. 6 and Table III).

## Discussion

BNP is a cardiac neurohormone that is secreted into the plasma from the ventricles in response to ventricular volume expansion and pressure overload (3). BNP plasma levels have been shown to be significantly higher in patients with decompensated chronic HF as compared with those in a control group (13). BNP levels provide an easy method for the early detection of HF and for assessing the severity of HF and the effectiveness of treatment (14). A number of previous studies have demonstrated that the levels of BNP and NT-proBNP are powerful prognostic markers across a spectrum of acute coronary syndromes (15), from unstable angina and non-ST elevation myocardial infarction to ST elevation myocardial infarction (16–18), as well as in patients with stable angina pectoris (19,20) and even in the absence of significant necrosis (21). BNP and NT-proBNP are present in human coronary arteries (22) and are associated with the extent and severity of coronary atherosclerotic lesions (23). The observations of the present study revealed a similar correlation; the proportion of patients with myocardial infarction was significantly higher in the BNP >100 pg/ml group as compared with the BNP ≤100 pg/ml group (P=0.039). Ischemia *per se* may function as a stimulus for the release of BNP and NT-proBNP (24). Overactivity of the sympathetic nervous system in the left ventricle appears to be an important mechanism for the induction of elevated BNP levels in chronic ischemic HF (4). BNP gene expression levels are upregulated in the ventricular wall by acute myocardial hypoxia, resulting in augmented plasma concentrations of BNP and proBNP (25,26).

NT-proBNP is independent of invasive measurements of LV function and the severity of coronary artery disease (20). The prognostic importance of BNP and NT-proBNP has been extensively studied in patients with HF, as well as in patients with acute coronary syndromes, with both markers having been demonstrated to be strong and independent predictors of morbidity and all-cause mortality (20,27,28). The predictors were also evident in the subgroup of patients with a LVEF of >60% and in patients with diabetes mellitus (29). A previous study (20) demonstrated that measuring NT-proBNP levels immediately prior to coronary angiography in patients with stable coronary heart disease provided prognostic information on all-cause mortality. The present study also demonstrated the same prognostic value. The incidence of cardiovascular mortality, readmission due to cardiovascular disease or mortality through other causes was significantly higher in the BNP >100 pg/ml group than in the BNP ≤100 pg/ml group. Kaplan-Meier analysis was performed to predict the survival times of the patients and the results indicated that the survival times were longer in the BNP ≤100 pg/ml group than in the BNP >100 pg/ml group. The results also demonstrated a negative correlation between the logBNP levels and the survival times of patients with cardiovascular disease, with survival times decreasing as the BNP levels increased. A plasma BNP level of 100 pg/ml was selected as a cut-off value for the prediction of cardiovascular morbidity and all-cause mortality, with a sensitivity of 57.44% and a specificity of 70.16% in all patients. BNP (or NT-proBNP) has been shown to have high negative predictive values as a single test (30). The observations of the present study revealed a similar outcome. The subjects of the present study included inpatients with various types of disease, including hypertension, diabetes, dyslipidemia, renal dysfunction and myocardial infarction. Therefore, this study demonstrates that the BNP level is correlated to the prediction of most cardiovascular diseases, not only one or several specific diseases. Thus, the application of BNP is wider than previously considered.

A previous study demonstrated that 40–50% of individuals with HF have a normal ejection fraction, and diastolic dysfunction is the presumed cause of diastolic HF (DHF) (31). Since abnormalities in diastolic function may not always produce symptoms of HF, the conditions are often missed and patients are predisposed to symptomatic HF due to the delay in treatment (31). Furthermore, the prognosis of patients suffering from DHF is as ominous as that of patients suffering from systolic HF. Diastolic dysfunction without symptoms (preclinical diastolic dysfunction) is common and is independently predictive of the future development of HF and cardiac mortality (32). Early diagnosis of LV diastolic dysfunction in an initial phase enables the start of effective treatment, which functions by stopping the progress of the disease and delaying the development of symptomatic HF (33). Analysis of the diastole by means of echocardiography, using Doppler measurements of transmitral and pulmonary vein blood flow velocities and tissue Doppler imaging, is widely accepted for clinical purposes (34). However, this type of assessment is expensive as it requires complex equipment, time-consuming as it involves the analysis of numerous variables and difficult as it must be performed by a skilled and trained operator (35). Thus, a simple and objective method to quantify diastole function with high sensitivity and specificity is required. An association between the levels of BNP and the indexes of diastolic function has been described in patients with reduced LVEF and in those with preserved LVEF (36). A previous study has shown that estimating BNP levels may be accepted as a fast and reliable blood test for the diagnosis of asymptomatic diastolic dysfunction. The BNP test may be used for the prediction of asymptomatic diastolic dysfunction in patients with hypertension (7). In addition, BNP levels may be used for the repeat evaluation of an occult LV dysfunction in patients who are periodically assessed for diabetic complications (8). Thus, BNP may be used as an adjunctive, reliable and objective method of estimating cardiac dysfunction in HCM (9). Measurement of BNP levels is simple and noninvasive, and can be easily and rapidly conducted in emergency departments to guide therapy, follow the response to therapy and predict the exercise capacity of patients (10). The observations of the present study indicated that the prognoses of patients with higher BNP levels were worse compared with those with lower BNP levels. A negative correlation between the levels of BNP and the survival times was identified in 395 HF patients with RLVSF; survival times of the HF patients with RLVSF decreased with increasing BNP levels. Furthermore, the predictive utility of plasma BNP levels in HF patients with RLVSF for determining the incidence of compound clinical endpoints was also demonstrated. Morbidity and mortality rates from cardiovascular diseases are increased in patients with high plasma BNP levels. However, a plasma BNP level cut-off value of 100 pg/ml may be used for the prediction of cardiovascular morbidity and all-cause mortality, with a sensitivity of 68.8% and a specificity of 45.93% in HF patients with RLVSF. The predictive utility of plasma BNP levels in HF patients with RLVSF is lower than in all the patients.

BNP has been shown to have a higher sensitivity (85 vs. 63%) and positive predictive value (69 vs 55%) than NT-proBNP. The negative predictive values of BNP and NT-proBNP were similar (70 and 71%, respectively) (37). The level of BNP appears to have a higher sensitivity and higher positive predictive value for the accurate diagnosis of severe LVSD than the level of NT-proBNP (38). The plasma half-life of BNP in humans is ~20 min, while the circulating half-life of NT-proBNP is ~120 min (38). Therefore, BNP levels may used to assess the current severity of LV dysfunction, guide therapy and follow the immediate response to therapy. However, NT-proBNP is unable to this since it has an assessment lag of ~10 h. Clearance of BNP is hypothesized to occur via two main mechanisms: Binding to clearance receptors and enzymatic degradation by the enzyme neutral endopeptidase (39). Clearance of NT-proBNP occurs predominantly via the kidney, thus, in patients with mild renal dysfunction, utility of diagnosis is seriously affected (40,41). Approximately 29% of HF patients have renal failure (42). BNP levels are a more useful diagnostic indicator for cardiogenic pulmonary edema than proBNP in patients aged ≥65 years (40). The estimated glomerular filtration rate has independent effects on the plasma BNP and NT-proBNP concentrations in patients with chronic kidney disease. However, NT-proBNP appears to be affected more than BNP by declining kidney function (43). Therefore, in the present study, the use of plasma BNP levels may have produced reliable, accurate and effective results.

There are several relevant limitations of the present study. Firstly, the number of patients in the study was small and the follow-up period was relatively short. Further studies with a larger number of patients that are conducted over a longer time period are required to assess the predictive value of BNP levels in patients with cardiovascular-related disease, particularly in patients with RLVSF. Secondly, the echocardiographic parameters should be interpreted with caution as the ejection fraction may be affected by different sections and atrial fibrillation. Further studies with myocardial perfusion imaging are required to calculate the LVEF, which is likely to provide more precise results. Thirdly, the sensitivity and specificity values for predicting the utility of plasma BNP levels in determining the incidence of compound clinical endpoints are not very high for either groups of patients. A combination of NT-BNP (or BNP) with LVEF has been shown to substantially improve the risk stratification for mortality, HF and new ischemic events (44).

In conclusion, the prognoses were worse for patients with higher levels of BNP. Furthermore, a significant correlation was observed between BNP levels and survival times in HF patients with RLVSF. BNP can predict the prognosis of patients with cardiovascular disease, particularly in HF patients with RLVSF.

## Figures and Tables

**Table I:** Baseline clinical characteristics according to the levels of BNP.

Characteristics	Patients with BNP ≤100 pg/ml (n=900)	Patients with BNP >100 pg/ml (n=515)	P-value
Age, years (range)	63 (50.0–76.0)	70 (56.4–83.6)	<0.05
Male gender, n (%)	513/900 (57.00)	277/515 (53.79)	0.242
Mortality, n (%)	7 (0.77)	24 (4.66)	<0.05
Readmission, n (%)	136 (15.11)	169 (32.82)	<0.05
Key events, n (%)	143 (15.89)	193 (37.47)	<0.05
Systolic dysfunction, n (%)	18/841 (2.14)	105/470 (22.34)	<0.05
Non-systolic dysfunction, n (%)	163/841 (19.38)	232/470 (49.36)	<0.05
Hypertension, n (%)	638 (70.89)	315 (61.16)	<0.05
Diabetes, n (%)	176 (19.55)	115 (22.33)	0.214
Dyslipidemia, n (%)	477 (53.00)	145 (28.15)	<0.05
Renal dysfunction, n (%)	31 (3.44)	84 (16.31)	<0.05
Myocardial infarction, n (%)	96 (10.67)	94 (18.25)	<0.05
Medication, n (%)			
β-blockers	480 (53.33)	250 (48.54)	0.083
Calcium antagonists	413 (45.89)	186 (36.12)	<0.05
Diuretics	230 (25.56)	378 (73.40)	<0.05
Nitrates	386 (42.89)	230 (44.66)	0.518
Antiplatelet agents	713 (79.22)	379 (73.59)	0.015
Statins	325 (36.11)	135 (26.21)	<0.05
ACEIs or ARBs	638 (70.88)	375 (72.82)	0.439
ACEIs	302 (33.56)	187 (36.31)	0.294
ARBs	336 (37.33)	188 (36.50)	0.756

BNP, brain natriuretic peptide; ACEIs, angiotensin converting enzyme inhibitors; ARBs, angiotensin receptor blockers.

**Table II:** Correlation between the logBNP levels and the incidence of compound endpoint events in all the patients.

	Compound endpoint events, n		
		
	Yes	No	Total
BNP >100 pg/ml	193	322	515
BNP ≤100 pg/ml	143	757	900
Total	336	1079	1415

BNP, brain natriuretic peptide.

**Table III:** Correlation between the logBNP levels and the incidence of compound endpoint events in the patients with diastolic dysfunction.

	Compound endpoint events, n		
		
	Yes	No	Total
BNP >100 pg/ml	86	146	232
BNP ≤100 pg/ml	39	124	163
Total	125	270	395

BNP, brain natriuretic peptide.
